# An Innovative Approach for Enhancing Bone Defect Healing Using PLGA Scaffolds Seeded with Extracorporeal-shock-wave-treated Bone Marrow Mesenchymal Stem Cells (BMSCs)

**DOI:** 10.1038/srep44130

**Published:** 2017-03-08

**Authors:** Youbin Chen, Jiankun Xu, Zhonglian Huang, Menglei Yu, Yuantao Zhang, Hongjiang Chen, Zebin Ma, Haojie Liao, Jun Hu

**Affiliations:** 1 Department of Orthopedics, First Affiliated Hospital, Shantou University Medical College, 57 Changping Road, Shantou, Guangdong 515041, China; 2 Department of Orthopaedics and Traumatology, Faculty of Medicine, the Chinese University of Hong Kong, Hong Kong SAR 999077, China; 3 Guangdong Provincial Key Laboratory of Malignant Tumor Epigenetics and Gene Regulation, Emergency Department, Sun Yat-Sen Memorial Hospital, Sun Yat-Sen University, Guangzhou, Guangdong 510120, China

## Abstract

Although great efforts are being made using growth factors and gene therapy, the repair of bone defects remains a major challenge in modern medicine that has resulted in an increased burden on both healthcare and the economy. Emerging tissue engineering techniques that use of combination of biodegradable poly-lactic-co-glycolic acid (PLGA) and mesenchymal stem cells have shed light on improving bone defect healing; however, additional growth factors are also required with these methods. Therefore, the development of novel and cost-effective approaches is of great importance. Our *in vitro* results demonstrated that ESW treatment (10 kV, 500 pulses) has a stimulatory effect on the proliferation and osteogenic differentiation of bone marrow-derived MSCs (BMSCs). Histological and micro-CT results showed that PLGA scaffolds seeded with ESW-treated BMSCs produced more bone-like tissue with commitment to the osteogenic lineage when subcutaneously implanted *in vivo*, as compared to control group. Significantly greater bone formation with a faster mineral apposition rate inside the defect site was observed in the ESW group compared to control group. Biomechanical parameters, including ultimate load and stress at failure, improved over time and were superior to those of the control group. Taken together, this innovative approach shows significant potential in bone tissue regeneration.

The repair of large bone defects resulting from trauma, congenital malformations, and surgical resection remains a challenge that is currently being addressed with the use of advanced tissue engineering approaches^1^. Currently, 2.2 million bone grafts are used annually worldwide^2^. Autografts and allografts are the major bone substitutes used to repair large bone defects. Autografts are considered the gold standard for bone defect repair but their application is restricted by limited bone quantities from harvest and donor-site morbidity^3^. Moreover, the amount of unsatisfactory repairs using autografts is as high as 30%^4^. Although allografts are readily available, osteogenesis is inhibited by immunogenic reactions from host tissues using this method^5^.

Bone graft substitute materials are used for a wide range of clinical applications. Three-dimensional-porous scaffolds of bone graft substitutes play a critical role in both cell targeting and transplantation strategies. These scaffolds provide surfaces that facilitate attachment, survival, migration, proliferation, and differentiation of stem/progenitor cells, as well as a void volume in which vascularization, new tissue formation, and remodeling can occur^6^. Poly(lactic-co-glycolic acid) [PLGA] is a substitute material that has been approved by the US Food and Drug Administration (FDA) for clinical application^7^. However, PLGA itself lacks osteo-inductivity. Although application of PLGA with osteoinductive factors, including bone morphogenetic protein (BMP)-2, vascular endothelial growth factor (VEGF), transforming growth factor (TGF)-β, and fibroblast growth factor-2, would significantly enhance bone defect repair, a single dose of an exogenous protein may not induce an adequate osteogenic signal, particularly in cases where host bone and surrounding soft tissue are compromised^8^,^9^,^10^,^11^. Therefore, finding a suitable strategy for enhancing bone defect healing with fewer complications is of great significance.

Regional gene therapy has been used to enhance bone repair, especially for treatment of fracture nonunion and spinal fusion. However, the transfer of genes encoding osteogenic proteins still associate with some biological risks which need to be demonstrated the safety before using in clinics^12^. Stem cell therapy has been used extensively for bone tissue engineering, however, when the cells were transplanted, most cells cannot escape apoptosis without which limits tissue repair^13^. Therefore, there is an urgent need to find additional interventions to better promote the curative effect of stem cells.

Extracorporeal shock-wave (ESW) therapy is a safe and effective alternative method for the treatment of delay-union or nonunion of long bone fractures^14^. A previous clinical study based on 72 patients with long bone fracture nonunion reported that the rate of bony union at was 40% at 3 months, 60.9% at 6 months, and 80% at 12 months^14^. Furthermore, ESW has been shown to elicit membrane perturbation, as well as Ras activation, resulting in the induction of nuclear osteogenic transcription factor activation, expression of collagen type I (Col1) and osteocalcin (OCN), and thus enhance terminal calcium nodule formation^15^. SW also stimulates expression of BMP, OCN, alkaline phosphatase (ALP), TGF-β1, and insulin-like growth factor genes, which promote the growth and differentiation of BMSCs towards osteoprogenitor cells *in vitro*^16^,^17^,^18^. More recently, our team found that ESW could promote the adhesion, spreading, and migration of osteoblasts via integrin-mediated activation of focal adhesion kinase (FAK) signaling^19^. It is well known that some of the physical processes of cues from the extracellular matrix (ECM) can influence stem cell fate, which is particularly relevant for the use of stem cells in bone tissue engineering^20^,^21^; however, to date, the potential of ESW in the regeneration of bone tissue has not been fully utilized.

Based on the findings from previous *in vitro* studies, we hypothesized that porous PLGA scaffolds seeded with ESW-treated BMSCs could significantly promote the repair of bone defects via similar mechanisms as observed *in vitro*. Our results suggest that this innovative approach may act as an alternative cost-effective treatment for the repair of bone defects.

## Results

### ESW promoted the proliferation of BMSCs

**Figure 1:**
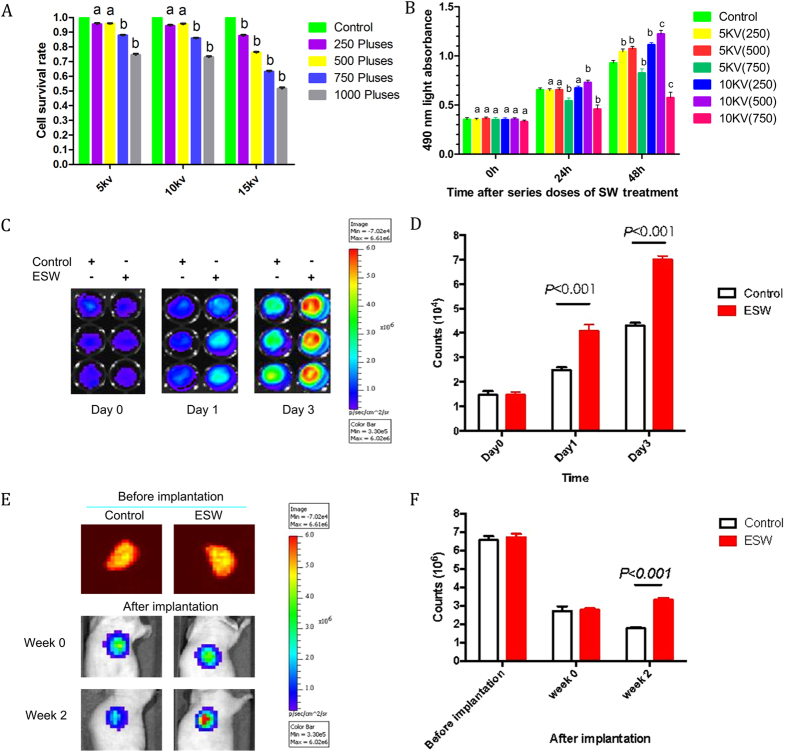
The optimal ESW intensity promoted proliferation of BMSCs. (**A**) Cell survival decreased with higher impulses of ESW, while no significant difference was observed below 500 impulses compared to control. With 5 KV or 10 KV for 250 or 500 impulses ESW treatment respectively, the cell survival was almost the same as control group (a, *P* > 0.05; b, *P* < 0.05 as compared to the control group by One-way ANOVA with Student–Newman-Keuls *post hoc* test). (**B**) MTT assay indicated that 500 impulses of 10 kV significantly augmented BMSC proliferation within 48 h after ESW treatment. Data are presented as the mean ± SD from triplicate experiments (a, *P* > 0.05; b, *P* < 0.05; c, *P* < 0.001 as compared to control group at the same period by One-way ANOVA with Student–Newman-Keuls *post hoc* test; n = 6). (**C**,**D**) The optimal ESW intensity promoted proliferation of GFP-BMSCs both *in vitro* and *in vivo*. GFP-BMSCs with or without ESW treatment were seeded with the same initial density (2,000 cells/well); cells from the ESW-treated group proliferated significantly faster than control cells at both time points (day 1 and 3 in culture; *P* < 0.001 by unpaired two-tailed Student’s t test, n = 3). (**D**,**E**) Dynamic fluorescence of control and ESW treated GFP-BMSCs (1.0 × 10^6^ cells) seeded onto PLGA scaffolds and subcutaneously implanted into nude mice was monitored using an IVIS 200 imaging system. Significantly more cells were retained *in vivo* in ESW group versus control at weeks 2 post-implantation (*P* < 0.001 by unpaired two-tailed Student’s t test, n = 3).

Differentiation of rat BMSCs into osteoblasts was verified by 1% Alizarin red S staining after being cultured for 2 weeks in osteogenic induction medium. BMSC differentiation into adipocytes was verified by 0.18% Oil Red O staining in adipogenic induction medium for 10 d, while differentiation into chondrocytes was verified by staining 5-mm BMSC sections with 0.05% Safranin O (Supplemental Fig. S1). Since ESW did not affect BMSC survival with energy up to 10 kV for 500 impulses, this dose was considered as optimal does and used for subsequent experiments (Fig. 1A and B).

Green fluorescent protein (GFP)-labeled BMSCs were used to further investigate whether ESW (10 kV, 500 pulses) could induce BMSCs proliferation using an IVIS 200 imaging system. Our data showed that ESW promoted BMSCs proliferation both *in vitro* and *in vivo* (Fig. 1C and D). A greater number of ESWT-treated cells were retained in scaffolds than control cells 2 weeks post-implantation (Fig. 1E and F).

### ESW enhanced the osteogenic differentiation of BMSCs

**Figure 2:**
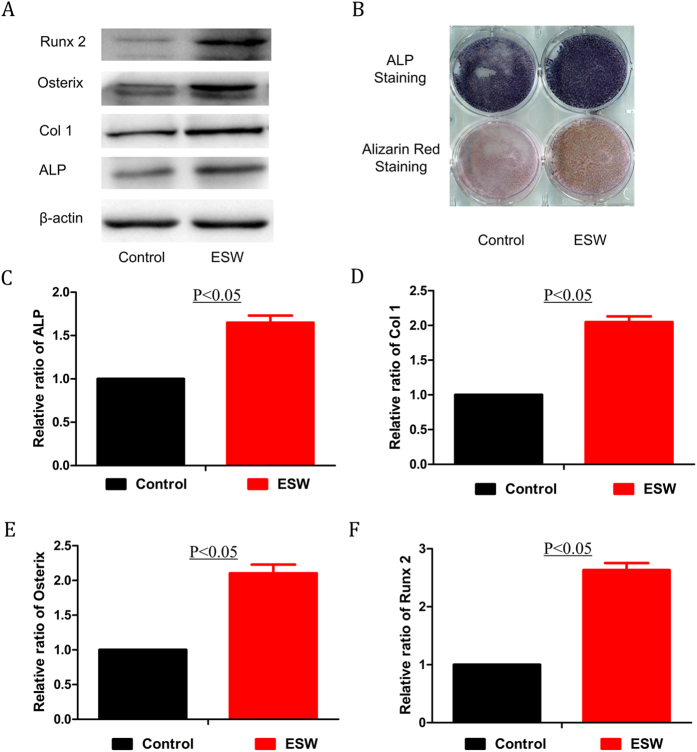
ESW promoted the osteogenic differentiation of BMSCs. (**A**) After ESW treatment, BMSCs were cultured for another 2 weeks in osteogenic induction medium and then proteins were extracted for western blotting. (**B**) Alizarin red and ALP staining also showed that ESW-treated BMSCs formed more calcium nodules and expressed higher levels of ALP. (**C**–**F**) Quantitative results showed that ESW significantly increased the expression levels of Col1, Runx2, osterix, and ALP (*P* < 0.05 by unpaired two-tailed Student’s t test).

Assessment of specific osteogenic transcription factor expression and calcium nodule formation 2 weeks post-ESW showed that the ESW group expressed higher levels of Col1, Osterix, Runx2, and ALP compared to control (Fig. 2A,C,D and F), further suggesting that ESW could induce differentiation of BMSCs into osteoblasts. Runx2 and Osterix are essential transcription factors that play important roles in the cell-fate decision through activation of cell type-specific genes which facilitate mesenchymal cells into becoming osteoblasts^22^. Enhancement of bone-mineralized matrix by ESW was demonstrated by an increase of calcium nodule formation in culture (Fig. 2B). These results indicate that BMSCs were committed to an osteogenic lineage and differentiated into mature osteoblasts and osteocytes post ESW treatment.

### ESW enhanced bone formation in nude mice

**Figure 3:**
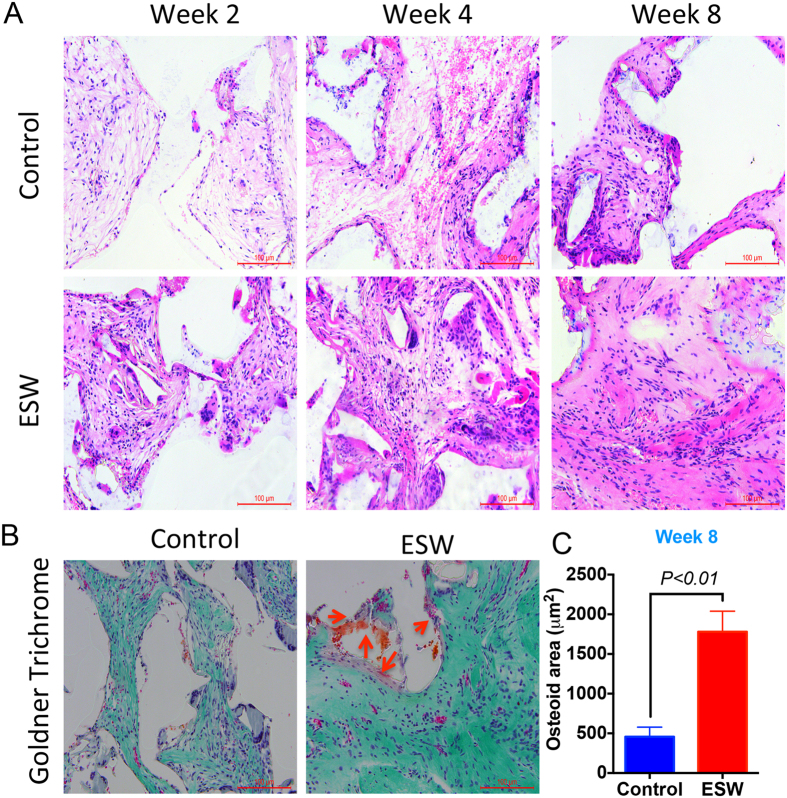
New bone formation is increased in PLGA scaffold seeded with ESW-treated BMSCs. (**A**) H&E staining showed that the tissues generated inside the pores of the scaffold became more solid as compared to those seeded with non-ESW-treated cells at the same time point. (**B**) Representative Goldner-Trichrome staining showed that significantly more osteoid (red arrows, purple to red) were produced 8 weeks post-ESW treatment. Corresponding quantitative data shows that significant difference in the total area of osteoid between these two groups (*P* < 0.01 by unpaired two-tailed Student’s t test). Error bars, mean ± SD, n = 5; scale bar, 100 μm.

There were more solid tissues formed in the ESW-treated group compared to the unconsolidated fibrous-like tissues formed in the control group (Fig. 3A). Goldner-Trichrome staining indicated that the ESW group produced significantly more osteoid in the surface of the newly formed tissue in transplants as compared to the control group (Fig. 3B and C).

**Figure 4:**
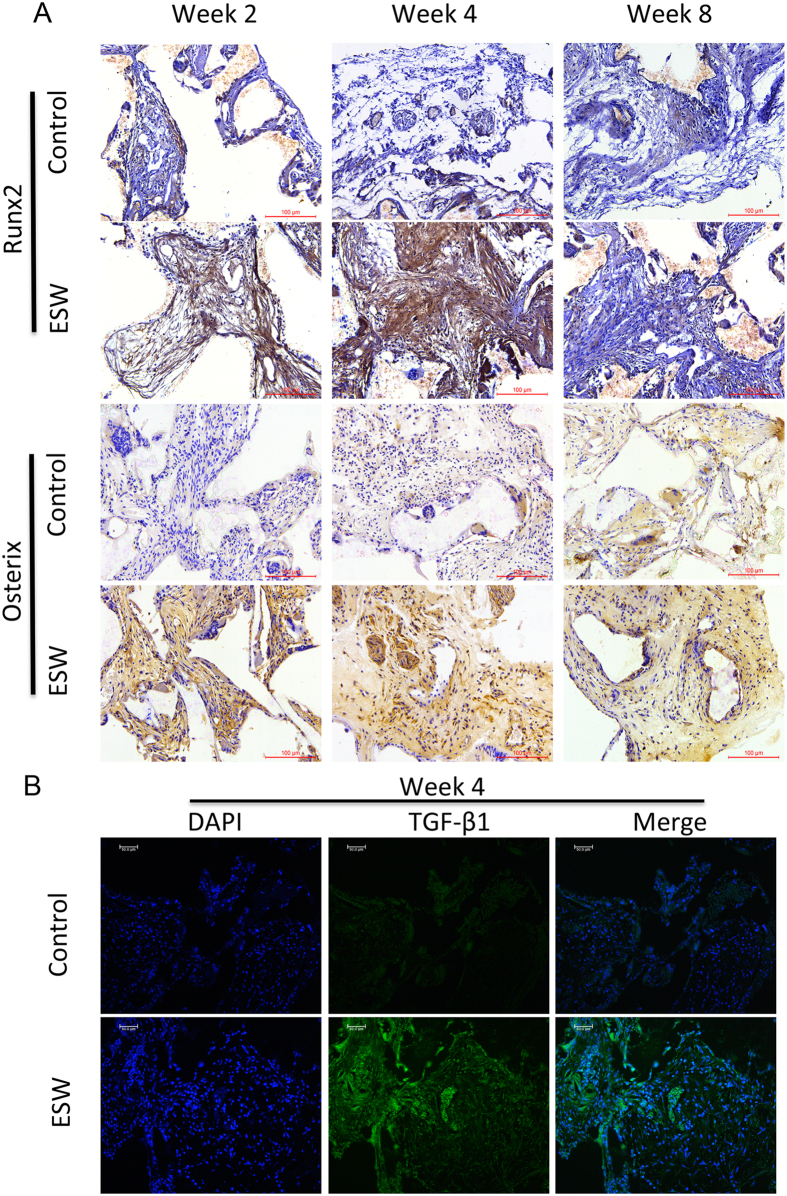
ESW treatment promoted the expression of Runx2, Osterix *in vivo*. (**A**) At 4 weeks post-ESW treatment, the sections had the strongest intensity, which indicated that there were more Runx2 (+), Osterix (+) cells in the ESW group. Scale bar, 100 μm; n = 5/group/time point. (**B**) At 4 weeks post ESW treatment, transplanted cells expressed more TGF-β1 (green) than the control group. The nuclei of the transplanted cells were stained with DAPI (blue). Scale bar, 50 μm; n = 5/group/time point.

Immunohistochemical staining showed more Osterix and Runx2 positive cells in newly formed bone matrix at all tested time points that obtained from nude mice samples (Fig. 4A). More importantly, we found more TGF-β1 positive cells inside the scaffolds in the ESW-treated group as compared to control group (Fig. 4B), indicating that a greater number of transplanted BMSCs from the ESW group were undergoing osteogenic differentiation^23^. It has previously been demonstrated that TGF-β1 is essential for bone remodeling and that TGF-β1 induces migration of BMSCs to the remodeling sites, which may attract more BMSCs to participate in bone regeneration^24^.

ESW-modified artificial bone form more new bone in the subcutaneously implanted nude mice. We applied micro-computed tomography (micro-CT) to access bone formation in PLGA scaffolds at selected post-operative time points. We found that ESW-modified artificial bone grew in the subcutaneously implanted nude mice with greater bone volume (BV), total tissue volume (TV), BV/TV, and bone mineral density (BMD) at both 4 weeks and 8 weeks post implantation (Table 1).

### ESW promoted new bone formation in rats and enhanced the biomechanical strength of regenerated bone

**Figure 5:**
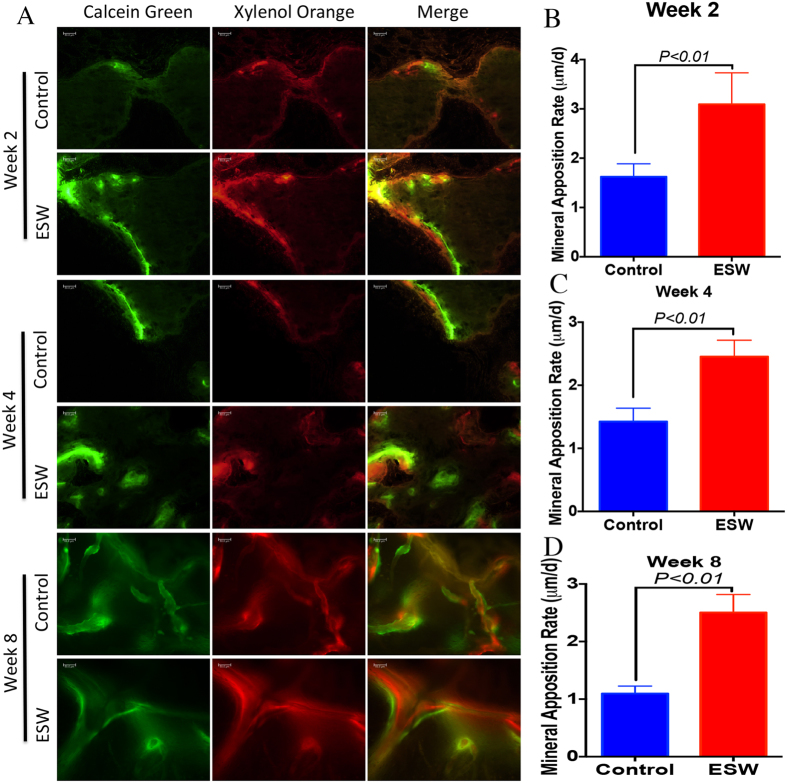
ESW promoted new bone formation in rats. (**A**) Fluorescent micrographs dynamically showed new bone formation in the defect site at 2, 4, and 8 weeks post-implantation, respectively. A more profound fluorescent deposition indicated a greater amount of new bone formation and remodeling in the ESW group over time. (**B**) Quantitative analysis of new bone within bone defect regions 2, 4 and 8 weeks post-implantation showed greater bone formation in the ESW group as compared to the control group. (**C**,**D**) Mineral apposition rate (MAR) was also significantly faster in ESW group as compared to control (*P* < 0.01 by unpaired two-tailed Student’s t test).

**Figure 6:**
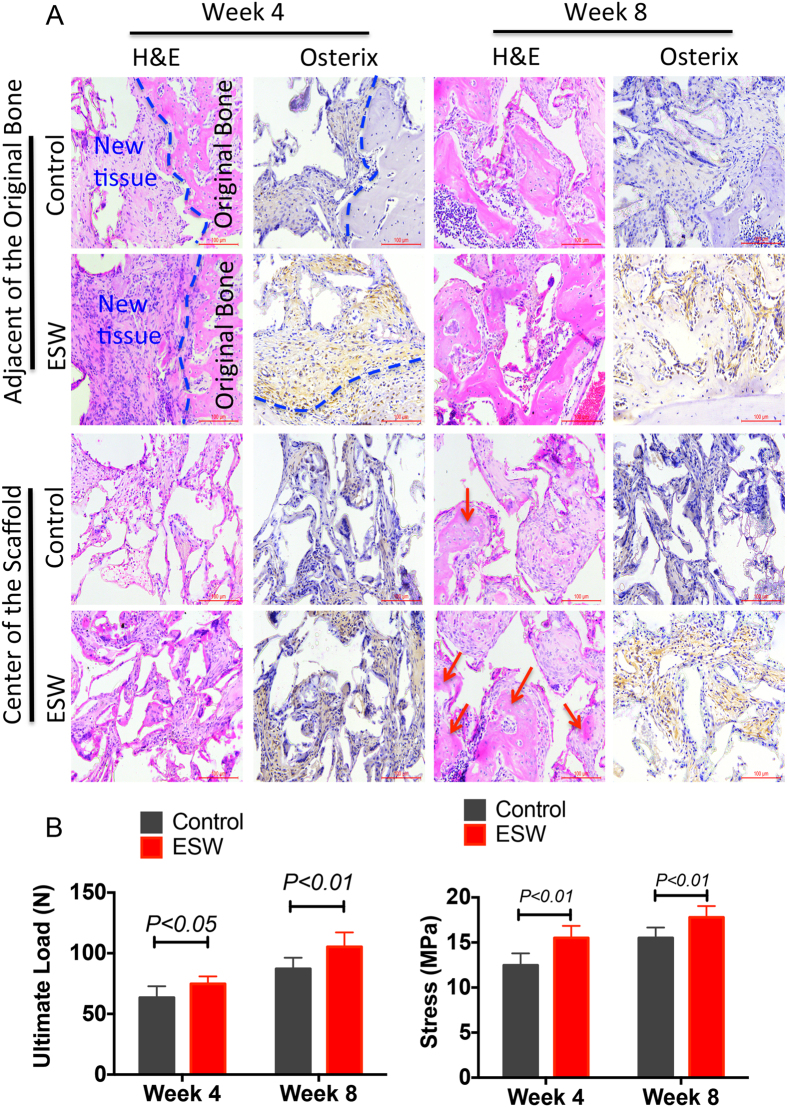
ESW promoted both biological and biomechanical repair of bone defect. (**A**) H&E stained sections 4 and 8 weeks post-implantation showed more newly formed bone in ESW scaffolds than in BMSC/PLGA without ESW treatment Immunohistochemistry of osteoblasts and mineralized sites 8 weeks post-implantation demonstrated that Osterix expression in newly formed bone had more and stronger signals within scaffolds, indicating more osteoblasts. New bone area was quantified 4 and 8 weeks post-implantation. In the ESW group, more newly formed bone was found in segmental defects compared with control groups. The ESW group had a larger area of newly formed bone. (**B**) Four-point bending biomechanical test was also performed to assess the properties of repaired bone tissues. ESW-treated artificial bone had stronger mechanical strength than the control group (*P* < 0.05 by unpaired two-tailed Student’s t test, n = 8).

Fluorescent microscopic evaluation revealed an increase in new bone formation, as indicated by a higher ratio of calcein green- to xylenol orange-labeled areas in the ESW group compared with controls at 2, 4, and 8 weeks (Fig. 5). Comparison of the amount of fluorescent labeling at all time points showed significantly higher of new bone area in the ESW group (*P* < 0.05). In order to further validate osteogenic differentiation of implanted BMSCs within bone defect sites, the key osteogenic marker, Osterix, was identified by immunohistochemical staining in decalcified paraffin sections. There were more osterix positive cells in newly formed bone matrix, both at the adjacent of original bone and center of the scaffold, at all tested time points (Fig. 6A).

Biomechanical strength recovery at the osteotomy site following scaffold implantation increased with healing time from week 4 to 8 (Fig. 6B). The ESW group had a significantly stronger strength at both 4 and 8 weeks post-implantation (*P* < 0.05, n = 8).

As presented in Table 2, ESW-modified artificial bone in mid-femur bone defect models showed greater BV, TV, BV/TV, and BMD, as compared to control group, at both 4 and 8 weeks post implantation. At week 8 post-implantation, the implanted region appeared to be better integrated and quite similar to the host bone, though transition interfaces were still discernable, in the ESW group (Fig. 6A).

## Discussion

The repair of large bone defects remains a challenge in clinical practice. Cell-based tissue engineering has created new and exciting opportunities with a broad array of potential clinical applications^6^. Mechanical stimulation led to a temporary increase in oxygen concentration at the site of injury. Combined biologic-free ferrogel and pressure cuff-driven mechanical compressions lead to enhanced muscle regeneration and muscle function compared with no-treatment controls, demonstrating the therapeutic potential of these mechanical interventions^25^. ESW has attracted particular attention in the field of *in vivo* bone tissue regeneration^26^,^27^ for its beneficial effects on cellular behaviors, such as proliferation^28^, differentiation^29^, adhesion, and migration^19^. Several previous experimental studies have demonstrated that ESW promotes bone healing by up-regulating bone growth factors and morphogenetic proteins^18^, as well as the activities of extracellular signal-regulated kinases, p38 kinase signaling, and Wnt/β-catenin signaling^19^,^30^. More importantly, we reveal a common underlying mechanism that inhibition of miR-138 by ESW significantly activate the FAK signaling with increased phosphorylation of FAK at tyr397 site. which triggers ERK1/2 signaling pathway and significantly promotes the osteogenic differentiation in human MSCs including tendon-derived stem cells, adipose-derived stem cells, and bone marrow mesenchymal stem cells (BMSCs)^31^.

In tissue engineering it is often desirable to pre-stimulated cell seeded constructs *in vitro*. In this study, we aimed to accelerate bone regeneration through the establishment of ESW-modified artificial bone. PLGA scaffolds seeded with BMSCs pretreated with an optimal dose of ESW (10 kV, for 500 impulses) have been identified as a useful approach for promoting bone defects repair. Using *in vitro* expansion, we can generate a large number of BMSCs that are crucial for forming new bone. Since MSC are unable to induce bone formation even though MSC are pre-committed in the osteogenic lineage in the absence of a scaffolding biomaterial^32^, the porous PLGA scaffolds are important to provide a surface and void volume for BMSCs to attach, proliferate, and differentiate^33^. Thus, in our study, porous PLGA scaffolds were structured with a ratio of 50:50, 80% porosity, and 250–500 μm pore size to provide an optimal environment for the formation of new bone and extracellular matrix^34^. Importantly, inductive stimuli are necessary to produce the desired tissue and using ESW as a biophysical stimulator has the potential for osteoinduction.

*In vitro* results demonstrated enhanced osteogenic differentiation and greater nodule formation after ESW. ESW also promoted the proliferation and differentiation of BMSCs towards osteoprogenitor cells, thereby facilitating bone regeneration, and inducing the expression of Runx-2, as well as the osteogenetic markers ALP, Col1, and Osterix in BMSCs during osteogenic induction. Runx2 plays a vital role in BMSC differentiation into osteoblast lineages by direct expression of ALP, Col1, matrix metalloproteinase-13, bone sialoprotein, osteopontin, and OCN genes^35^,^36^. We previously found that ESW treated-BMSCs dramatically increased protein levels of both the phosphorylated and total Runx-2 (ref. 31). The cells must enter the late stage of osteogenesis and express Osterix, which are controlled by Runx2 to deposit calcium^37^; Osterix is downstream of Runx2 in mesenchymal cells^38^. All these are strong evidence support the role of ESW in mediating the osteogenic differentiation of BMSCs.

ESW increased BMP-2 expression, as well as ALP activity and calcium deposits with respect to untreated adipose-derived stem cells^39^. PCL scaffolds delivery of rhBMP-2 released in a sustained manner exhibit good cellular activity for both cell proliferation and osteogenic activity^40^. Major advances in bone tissue engineering with scaffolds have been achieved through the use of growth factors, drugs, and gene delivery systems targeting osteogenic differentiation, though with some drawbacks^8^,^9^,^10^,^11^. Given ESW also increases the expression of BMP-2 in BMSCs, as an alternative approach, ESW provides a safe, cost-effective, and rational strategy for promoting osteogenic differentiation.

Previous *in vivo* studies have shown that greater amounts of new bone are detected at 2, 4, and 8 weeks after implantation of ESW-treated BMSCs. In this study, decalcified histology confirmed that there were significantly more Osterix positive cells in the newly generated tissue in ESW group, indicating that a higher proportion of the transplanted BMSCs were under-differentiating towards osteoblastic lineage. For bone defect repair, it is beneficial if greater stem cells differentiate into bone-forming osteoblasts. In femur bone defect, the implantation was integrated into the existing tissue, resulting in a seamless transition in the interface. After a 4-week subcutaneous implantation in nude mice, we detected that the ESW group expressed higher levels of TGF-β1, which plays an important role in bone regeneration and remodeling^41^. Active TGF-β1 release also induces migration of BMSCs to the bone absorptive sites that are mediated through SMAD signaling pathway^24^.

Previous *in vitro* studies have shown that the addition of TGF-β1 could increase mRNA levels of osteoblast differentiation markers (*Runx2, OPN* and *Col1*) and reduce self-renewal markers (*Oct4, Stella, Nanos3*, and *Abcg2*)^42^. On the surface of the newly formed tissue, we found significantly greater osteoid produced in the ESW group.

In this study, we confirmed that ESW promoted healing of bone defects that facilitate new bone tissues to form in the defect site. However, there are also some limitations to our study. First, as a proof-of-concept study, our research did not use large animals as models in this experiment. Since our research explored the possibility of the shock wave application in tissue engineering for the first time, we chose a partial bone defect model in the mid-shaft of the rat femur, which did not require plate-screw internal fixation. Therefore, in our *in vivo* experiments, the scaffolds used are in quite a small size (5-mm in length). We will carry out large animal (dogs or goats) *in vivo* studies to further investigate the efficacy of this innovative approach in the future. In a previous preclinical study, scaffolds with a diameter of 4-mm and a height of 16-mm were used in New Zealand rabbits^43^. In addition, a critical-sized segmental bone defect created in the mid-portion of the femoral diaphysis of adult dog was repaired by using MSCs loaded onto a hollow ceramic cylinder consisting of TCP-HA with a length of 21-mm^44^. In a clinical study published in 2001, expanded BMSCs were placed on macro-porous HA scaffold to successfully treated a patient with 70-mm segmental defect of humerus^45^. All these evidence suggest the translational potential of our current finding for treating larger bone defect. However, as we known, the transplanted cells need to access the nutrients (oxygen, glucose, and amino acids) and to clear the metabolism (CO_2_, lactates, and urea). Micro-vessel formation within the scaffold is the prerequisite for cell survival after implanted. Of note, shock wave treatment can enhance the expression of VEGF *in vitro* and *in vivo*^46^,^47^,^48^, which is an important factor for vessel formation. Nevertheless, regeneration of bone tissue in larger scaffolds (for example, 2.0 × 2.0 × 6.0 inch^3^) remains a great challenge for clinicians, as the inner BMSCs would apoptosis without sufficient nutrients. Though our currently developed scaffold is not ready to be implanted in humans, many available advanced bio-techniques (such as 3D bio-printing and bioreactors) make it possible to better incorporate the cells into the scaffolds with larger size^49^. We will be also able to fabricate many small blocks of artificial bone tissues using the bioreactors in quite a short period^49^. These small blocks may fully fill the demand to repair larger bone defect. Second, we still need to investigate if β-tricalcium phosphate or hydroxyapatite (β-TCP or HA) scaffolds with intrinsic osteo-inductivity seeded with shock wave treated BMSCs would further enhance bone defect repair. As the cells have received shock wave treatment before seeding on the scaffolds, rather than using shock wave to treat the scaffolds with cells, it is likely that the positive results observed from the cells seeded on PLGA scaffold can be extrapolated and applied to BMSCs seeded on other scaffolding materials. Third, it is possible that other signaling pathways are involved in the enhancement of bone defect healing induced by the current approach. In our previous study, we found that ESW-induced expression of integrin α5β1 after 3 h post-treatment significantly increased β-catenin expression^19^. The elevation of β-catenin in stem cells can suppress PPARγ expression, which may result in sufficient specific differentiation into osteoblasts^50^.

In conclusion, our current study used PLGA composited with allograft BMSCs pretreated with ESW for the repair of mid-femur defects in rats and investigated its ability to form new bone in nude mice. These results demonstrated that ESW may promote the proliferation and osteogenic differentiation of BMSCs *in vitro*. Combination of PLGA scaffolds and cells treated with ESW produced a greater amount of bone regeneration when treating femur bone defect, as compared to the group receiving PLGA seeded with cells without ESW treatment. The results discussed within this article summarize the potential benefits of using ESW for controlling the differentiation of BMSCs. Since there is a significant need for reliable tissue models within the clinical and pharma industries, the control of cell behavior and stem cell differentiation would be highly beneficial. This technique provides significant potential benefits over existing technologies, as cellular responses can be initiated without the use of expensive chemical induction factors and complex fabrication procedures. Therefore, this innovative approach may act as an alternative and cost-effective treatment for bone defect repair.

## Methods

### Experimental animals

For cell isolation and culture, ten 4-week-old male Sprague-Dawley (SD) rats were obtained from the Experimental Animal Center of Shantou University Medical College (Shantou, China). The Animal Research Ethics Committee of Shantou University Medical College approved all relevant experiments in this study. Care of rats in this investigation aligned with the National Institutes of Health guidelines (National Institutes of Health (1996) *Guide for the Care of Use of Laboratory Animals*, NIH Publication 85–23, National Institutes of Health, Bethesda, MD). All methods were performed in accordance with the relevant guidelines and regulation.

### Reagents and antibodies

Low/high glucose Dulbecco’s minimal essential medium (LG-DMEM, HG-DMEM), α-MEM and fetal bovine serum (FBS) were purchased from Hyclone. Transforming growth factor β3, dexamethasone, ascorbic acide2-phosphate, and bone morphogenetic protein-2 was purchased from Peprotech. BD™ ITS Premix was from BD Biosciences. Xylenol orange and calcein green was purchased from Sigma-Aldrich. For western blotting, rabbit anti-Runx2 (ab102711), Col1(ab34710), Osterix (ab22552), and ALP (ab95462) primary antibody were purchased from Abcam. Anti-β-actin antibody (sc-130657) and ALP-conjugated goat anti-rabbit IgG secondary antibody (sc-2004) were from Santa Cruz. Antibody detection was carried out using a BCIP/NBT kit (Zymed, Invitrogen). Other chemicals and reagents were of molecular biology grade and were purchased from local commercial stores.

### Culture and identification of BMSCs

Isolation and expansion of BMSCs were performed according to a previously described protocol^51^. Ten male SD rats (4 weeks old) were sacrificed and bone marrow was harvested by flushing femoral and tibial cavities with α-Modified Eagle’s Medium (α-MEM). Cells collected from bone marrow were seeded at a density of 1.0 × 10^6^ cells/mL in culture flasks with α-MEM containing 10% FBS and 1% penicillin-streptomycin and incubated in a 5% CO_2_ humidified atmosphere at 37 °C. Non-adherent cells were removed after 3 d in culture and fresh culture medium was added; the culture medium was changed every 3 d. Cells were passaged when they reached approximately 90% confluence. Cells obtained at in passage 3–5 were used for further analyses.

Cells derived from rat bone marrow were authenticated based on three known BMSC attributes^52^; in particular, multi-lineages differentiation potential *in vitro* under controlled conditions. To confirm the multi-lineages differentiation potential, sub-cultured cells were induced to differentiate into osteoblasts, adipocytes, and chondrocytes using procedures reported by Pittenger *et al*.^53^ and Tsutsumi *et al*.^54^. The osteogenic media include DMEM with 10% FBS supplemented 50 mg/mL Vitamin C, 10 mM β-glycerophosphate, and 10 nM dexamethasone (Sigma Aldrich); adipogenic media were DMEM with 10% FBS supplemented 500 mM isobutylmethyl xanthine, 1 mM dexamethasone, 10 mg/mL insulin, and 200 mM indomethacin. Chondrogenic media were serum-free DMEM supplemented with 1% BD™ ITS Premix (BD Biosciences, Franklin Lakes, NJ, USA; consisting of 6.25 mg/mL insulin, 6.25 mg/mL transferrin, 6.25 ng/mL selenous acid, 5.33 mg/mL linoleic acid, and 1.25 mg/ml bovine serum albumin), 10 ng/mL transforming growth factor β3 (TGF-β3; Peprotech, Rocky Hill, NJ, USA), 100 nM dexamethasone, 50 mg/mL ascorbic acide2-phosphate, and 500 ng/mL bone morphogenetic protein 2(BMP-2; Peprotech).

### ESW treated of rat BMSCs *in vitro*

ESW was generated using Huikang type IV ESW equipment (Huikang, Shengzhen, China). BMSCs were subjected to ESW as previously described^19^. Cells (1.0 × 10^6^ cells/ml) were suspended in 15-ml sterile polystyrene tubes and 250, 500, 750, and 1000 impulses of 10 kV ESW were applied to identify the optimal intensity required. Each treatment lasted 10 min. After ESW treatment, cells were cultured for 24 and 48 h for MTT proliferation assay after assessing cell survival after 1 h with 0.4% trypan blue. Once an optimal impulse of ESW was determined, ESW-treated cells were placed onto plastic dishes or culture plates evaluation of multi-lineage differentiation potential per protocol. BMSCs without ESW treatment were used as controls.

### Alizarin Red and ALP staining

The degree of calcium deposition was determined by Alizarin red staining. After 21 d, the culture medium was removed and cells were washed with distilled water and fixed with 70% ethanol, finally stained with 1% Alizarin red S in distilled water (pH 4.2) for 10 min. ALP staining was performed to examine osteoblast differentiation using an ALP staining kit (NCIP/NBT Alkaline Phophatase Color Development Kit, PanEra, AAPR279) according to the manufacturer’s instructions.

### Western blotting

Cells were seeded onto 10-cm dishes. total protein lysates were extracted in ice-cold radioimmunoprecipitation assay buffer and sonicated twice for 6 s each^19^. Separation of Triton X-100-soluble and -insoluble fractions was performed according to a previous protocol. Protein concentrations were determined using a BCA protein assay kit. Protein lysates (100 mg) were separated on a 10% Bis–Tris polyacrylamide gel and subsequently transferred onto a polyvinylide fluoride membrane (Amersham Pharmacia Biotech, Buckinghamshire, UK). Membranes were blocked with 5% non-fat milk in Tris-buffered saline containing 0.1% Tween-20 and probed with a rabbit anti-Runx2 (1:1000; Abcam ab102711), Col1(1:1000; Abcam ab34710), Osterix (1:1000; Abcam ab22552), and ALP (1:1000; Abcam ab95462) primary antibody followed by ALP-conjugated goat anti-rabbit IgG secondary antibody (1:10000; Santa Cruz sc-2004). Antibody detection was carried out using a BCIP/NBT kit (Zymed, Invitrogen) according to the manufacturer’s instructions. As a loading control, membranes were immunoblotted with a rabbit anti-β-actin antibody (1:1000; Santa Cruz sc-130657). Protein levels were quantified using ImageJ software (National Institutes of Health, MD, USA). After correcting for background, the integrated density of each protein band was measured using the same selection surface for each measurement^19^.

### Animals surgical procedures and experimental design

Ninety-four male SD rats and ten nude mice were obtained from the Experimental Animal Center of Shantou University Medical College (Shantou, China). Three 8-week-old male nude mice were used as a GFP marker group to observe the BMSCs survival and seven 8-week-old male nude mice were used to observe the osteogenic differentiation of ESW-modified BMSC *in vivo*; 12-week-old male SD rats were used for implantation as hosts. To evaluate the effects of ESW on the quality and healing time of the formation of bone tissue of male SD rats, 94 male SD rats were randomly assigned into two different treatment groups. All surgical procedures were performed under general anesthesia by intraperitoneal injection of 1.5% pentobarbital. After surgery (see methods below), all experimental animals were housed under pathogen-free conditions (2–4 per cage) that allowed them free access to food pellets and tap water.

### Subcutaneous implantation in nude mice

We investigated bone defect regeneration using BMSCs grown in 3D-PLGA scaffolds. PLGA (L/G ratio 50:50, MW 40,000-75,000) scaffolds with 80% porosity (average diameter of 250–500 μm) were produced by Shanghai Biotech. Ltd based on the principles recommended in a previous study^33^. BMSCs were pretreated with or without ESW treatment. We subcutaneously transplanted BMSCs (1.0 × 10^6^ cells) grown on PLGA scaffolds (5.0 × 5.0 × 5.0 mm^3^) pretreated with or without ESW treatment into nude mice. A 3 cm longitudinal incision was made overlaying the spine and four subcutaneous pockets were made using blunt dissection. One scaffold was implanted in each pocket and then the wound was closed. Mice were sacrificed at 2, 4, and 8 weeks post-implantation and repaired tissue was assessed both macroscopically and histologically.

### Mid-femur bone defects in rats

Defects were created surgically on the mid-femur of rats^55^. A 5.0 mm wide and approximately one third of cross-section diameter of the femur in depth defect was made in mid-shaft of femurs by columnar dental diamond burs to all SD rats. Subsequently, the defecting areas were filled either with ESW treatment cell-aggregate or cell-aggregate randomly. Rats were sacrificed at 2, 4, and 8 weeks post-implantation and repaired tissues were assessed both macroscopically and histologically.

### Sequential fluorescent labeling and sample harvesting

Sequential fluorescent labeling was used to study bone dynamic remodeling within segmental bone defects using an established protocol^56^,^57^,^58^. In brief, xylenol orange (90 mg/kg body weight; Sigma-Aldrich GmbH) and calcein green (10 mg/kg body weight; Sigma-Aldrich GmbH) were injected subcutaneously and sequentially into rats (n = 5/group) at 10 and 3 days before euthanasia, respectively.

### CT evaluation of new bone formation

At 4 and 8 weeks post-implantation, newly formed bone was evaluated using microCT 40 (Scanco Medical, Brüttisellen, Switzerland) according to a published protocol^58^,^59^. Briefly, the entire defect region was scanned at a spatial resolution of 15 μm and the bony compartment was segmented from the marrow and soft tissue for subsequent analyses using a global threshold procedure. A threshold equal to or above 150 represented bony tissue; a threshold below 150 represented bone marrow, soft tissue, and implanted composite scaffolds^60^. New bone formed within the bone defect region was acquired for quantification of bone mineral density, tissue volume, and bone volume (n = 8/group/time point).

New bone formed in the nude mice after subcutaneous transplant was defined as a threshold equal to or above 100 represented bony tissue; a threshold below 100 represented soft tissue and implanted composite scaffolds (n = 5/group/time point).

### Histological analysis

Harvested femora from rats and PLGA scaffolds from nude mice were dissected free from soft tissue, fixed in 10% buffered formalin for 24 h, decalcified with 10% EDTA (pH 7.4) for 4 weeks, dehydrated in a gradient series of ethanol, embedded in paraffin, and cut through the long axis into 5-mm-thick sections. Sections were stained with hematoxylin and eosin (H&E), and immunostained for Runx-2 and Osterix expression. In brief, sections were incubated with proteinase K (Dako, Glostrup, Denmark) for 6 min at room temperature for antigen retrieval, followed by blocking with 1% H_2_O_2_/methanol (Sigma-Aldrich) for 30 min with primary antibodies diluted 1:200 with antibody diluent (Dako), rabbit polyclonal anti-Runx-2 (ab102711, Abcam), and rabbit polyclonal anti-Osterix (ab22552, Abcam). Secondary antibody incubation was performed using EnVision™ (Dako) for 30 min. The colorimetric reaction was developed with diaminobenzidine (Dako) and counterstained with hematoxylin.

Part of the samples harvested from rats and nude mice were prepared for undecalcified histology. Goldner-Trichrome staining was performed for static histomorphometric measurement. A semi-automatic digitizing image analysis system (OsteoMetrics, Atlanta, GA, USA) was used to calculate the formation of osteoid.

### Biomechanical test

Harvested femurs were prepared for biomechanical testing by removing as much soft tissue as possible, followed by immediate wrapping in gauze dampened with physiological saline to prevent dehydration. Tests were conducted within minutes of harvest. Femora were subjected to destructive four-point bending in the anteroposterior direction using a Uniaxial Mechanical Testing Machine (H25KM, Tinius Olsen). Femurs were placed on the lower supports on their posterior surface, with one support under the trochanter major and the other under the distal end. The distance between the two supports was set at 20 mm and femurs were carefully positioned so that the midpoint between the two bone-implant interfaces was aligned at the center point between the two supports. In order to prevent twisting of the bone during loading, the intercondylar fossa was gently pressed onto the bending apparatus. Before actual testing, a small stabilizing preload (10 N) was applied, after which the specimen was loaded to failure at a crosshead speed of 1.0 mm/min. The load at failure was recorded as the bending strength.

### Statistical analyses

Data are presented as mean ± standard deviation (SD). Unpaired two-tailed Student’s t test was used for comparison between two groups. One-way ANOVA with Student–Newman-Keuls *post hoc* test was used for multiple-group comparisons. Differences with a *P* < 0.05 were regarded as significant.

## Additional Information

**How to cite this article**: Chen, Y. *et al*. An Innovative Approach for Enhancing Bone Defect Healing Using PLGA Scaffolds Seeded with Extracorporeal-shock-wave-treated Bone Marrow Mesenchymal Stem Cells (BMSCs). *Sci. Rep.* **7**, 44130; doi: 10.1038/srep44130 (2017).

**Publisher's note:** Springer Nature remains neutral with regard to jurisdictional claims in published maps and institutional affiliations.

## Supplementary Material

Supplementary Information

## Figures and Tables

**Table 1:** Comparison of Micro-CT Analysis of Bone Formation in PLGA Scaffolds Subcutaneously Implanted into Nude Mice Across Time Points.

	Week 4		P value	Week 8		P value
	Con	ESW		Con	ESW	
BV (mm^3^)	1.13 ± 0.11	1.55 ± 0.27	*P* < *0.01*	1.83 ± 0.25	2.44 ± 0.24	*P* < *0.01*
TV (mm^3^)	5.39 ± 1.26	5.74 ± 1.08	*P* > *0.05*	6.13 ± 1.41	5.92 ± 0.95	*P* > *0.05*
BV/TV	0.22 ± 0.05	0.28 ± 0.03	*P* < *0.05*	0.31 ± 0.08	0.40 ± 0.06	*P* < *0.05*
B. Th (mm)	0.06 ± 0.02	0.10 ± 0.03	*P* < *0.05*	0.09 ± 0.03	0.15 ± 0.02	*P* < *0.01*
B. Sp (mm)	0.64 ± 0.18	0.48 ± 0.15	*P* < *0.01*	0.55 ± 0.14	0.39 ± 0.11	*P* < *0.01*
BMD (mg HA/cm^3^)	341.75 ± 15.63	418.52 ± 17.42	*P* < *0.01*	368.72 ± 21.38	459.66 ± 20.03	*P* < *0.01*

Values are mean ± SD; n = 5/group/time point; comparisons were made between groups at the same time point by unpaired two-tailed Student’s t test.

**Table 2:** Comparison of Different Parameters in Micro-CT Analysis From Rat Bone Defect Model Across Time Points.

	Week 4		P value	Week 8		P value
	Con	ESW		Con	ESW	
BV (mm^3^)	6.09 ± 0.93	8.84 ± 1.07	*P* < *0.01*	8.25 ± 1.41	12.71 ± 1.19	*P* < *0.01*
TV (mm^3^)	20.51 ± 2.52	21.71 ± 3.04	*P* > *0.05*	25.38 ± 3.44	24.72 ± 3.17	*P* > *0.05*
BV/TV	0.32 ± 0.07	0.39 ± 0.04	*P* < *0.05*	0.33 ± 0.05	0.48 ± 0.05	*P* < *0.01*
B. Th (mm)	0.14 ± 0.03	0.18 ± 0.02	*P* < *0.01*	0.15 ± 0.05	0.24 ± 0.03	*P* < *0.01*
B. Sp (mm)	0.33 ± 0.07	0.25 ± 0.05	*P* < *0.05*	0.29 ± 0.10	0.18 ± 0.09	*P* < *0.01*
BMD (mg HA/cm^3^)	448.33 ± 31.63	607.53 ± 37.82	*P* < *0.01*	483.75 ± 25.84	782.15 ± 37.69	*P* < *0.01*

Values are mean ± SD; n = 8/group/time point; comparisons were made between groups at the same time point by unpaired two-tailed Student’s t test.

## References

[b1] Vacanti J. P. & Langer R. Tissue engineering: the design and fabrication of living replacement devices for surgical reconstruction and transplantation. Lancet 354 Suppl 1, SI32–34 (1999). 10437854 10.1016/s0140-6736(99)90247-7

[b2] Calori G. M., Mazza E., Colombo M. & Ripamonti C. The use of bone-graft substitutes in large bone defects: any specific needs? Injury 42 Suppl 2, S56–63 (2011). 21752369 10.1016/j.injury.2011.06.011

[b3] Virk M. S. . Influence of short-term adenoviral vector and prolonged lentiviral vector mediated bone morphogenetic protein-2 expression on the quality of bone repair in a rat femoral defect model. Bone 42, 921–931 (2008). 18295562 10.1016/j.bone.2007.12.216

[b4] Gamradt S. C. & Lieberman J. R. Genetic modification of stem cells to enhance bone repair. Ann Biomed Eng 32, 136–147 (2004). 14964729 10.1023/b:abme.0000007798.78548.b8

[b5] Gazdag A. R., Lane J. M., Glaser D. & Forster R. A. Alternatives to Autogenous Bone Graft: Efficacy and Indications. J Am Acad Orthop Surg 3, 1–8 (1995). 10790647 10.5435/00124635-199501000-00001

[b6] Muschler G. F., Nakamoto C. & Griffith L. G. Engineering principles of clinical cell-based tissue engineering. J Bone Joint Surg Am 86-A, 1541–1558 (2004). 15252108 10.2106/00004623-200407000-00029

[b7] Hutmacher D. W. Scaffolds in tissue engineering bone and cartilage. Biomaterials 21, 2529–2543 (2000). 11071603 10.1016/s0142-9612(00)00121-6

[b8] He F. . Improving bone repair of femoral and radial defects in rabbit by incorporating PRP into PLGA/CPC composite scaffold with unidirectional pore structure. J Biomed Mater Res A 103, 1312–1324 (2015). 24890626 10.1002/jbm.a.35248

[b9] Goshima K., Nakase J., Xu Q., Matsumoto K. & Tsuchiya H. Repair of segmental bone defects in rabbit tibia promoted by a complex of beta-tricalcium phosphate and hepatocyte growth factor. J Orthop Sci 17, 639–648 (2012). 22763716 10.1007/s00776-012-0262-4

[b10] Hollinger J. O. . Accelerated fracture healing in the geriatric, osteoporotic rat with recombinant human platelet-derived growth factor-BB and an injectable beta-tricalcium phosphate/collagen matrix. J Orthop Res 26, 83–90 (2008). 17676626 10.1002/jor.20453

[b11] Street J. . Vascular endothelial growth factor stimulates bone repair by promoting angiogenesis and bone turnover. Proc Natl Acad Sci USA 99, 9656–9661 (2002). 12118119 10.1073/pnas.152324099 PMC124965

[b12] Bonadio J. Tissue engineering via local gene delivery. J Mol Med (Berl) 78, 303–311 (2000). 11001527 10.1007/s001090000118

[b13] Hwang N. S. . *In vivo* commitment and functional tissue regeneration using human embryonic stem cell-derived mesenchymal cells. Proc Natl Acad Sci USA 105, 20641–20646 (2008). 19095799 10.1073/pnas.0809680106 PMC2634917

[b14] Wang C. J., Chen H. S., Chen C. E. & Yang K. D. Treatment of nonunions of long bone fractures with shock waves. Clin Orthop Relat Res 95–101 (2001). 10.1097/00003086-200106000-00013 11400901

[b15] Wang F. S. . Physical shock wave mediates membrane hyperpolarization and Ras activation for osteogenesis in human bone marrow stromal cells. Biochem Biophys Res Commun 287, 648–655 (2001). 11563844 10.1006/bbrc.2001.5654

[b16] Takahashi K. . Gene expression for extracellular matrix proteins in shockwave-induced osteogenesis in rats. Calcif Tissue Int 74, 187–193 (2004). 14595530 10.1007/s00223-003-0043-3

[b17] Wang C. J. . The effects of shockwave on bone healing and systemic concentrations of nitric oxide (NO), TGF-beta1, VEGF and BMP-2 in long bone non-unions. Nitric Oxide 20, 298–303 (2009). 19281856 10.1016/j.niox.2009.02.006

[b18] Wang F. S., Yang K. D., Chen R. F., Wang C. J. & Sheen-Chen S. M. Extracorporeal shock wave promotes growth and differentiation of bone-marrow stromal cells towards osteoprogenitors associated with induction of TGF-beta1. J Bone Joint Surg Br 84, 457–461 (2002). 12002511 10.1302/0301-620x.84b3.11609

[b19] Xu J. K. . Optimal intensity shock wave promotes the adhesion and migration of rat osteoblasts via integrin beta1-mediated expression of phosphorylated focal adhesion kinase. J Biol Chem 287, 26200–26212 (2012). 22654119 10.1074/jbc.M112.349811 PMC3406705

[b20] Guilak F. . Control of stem cell fate by physical interactions with the extracellular matrix. Cell Stem Cell 5, 17–26 (2009). 19570510 10.1016/j.stem.2009.06.016 PMC2768283

[b21] Yim E. K., Darling E. M., Kulangara K., Guilak F. & Leong K. W. Nanotopography-induced changes in focal adhesions, cytoskeletal organization, and mechanical properties of human mesenchymal stem cells. Biomaterials 31, 1299–1306 (2010). 19879643 10.1016/j.biomaterials.2009.10.037 PMC2813896

[b22] Sinha K. M. & Zhou X. Genetic and molecular control of osterix in skeletal formation. J Cell Biochem 114, 975–984 (2013). 23225263 10.1002/jcb.24439 PMC3725781

[b23] Shakir S. . Transforming growth factor beta 1 augments calvarial defect healing and promotes suture regeneration. Tissue Eng Part A 21, 939–947 (2015). 25380311 10.1089/ten.tea.2014.0189 PMC4356478

[b24] Tang Y. . TGF-beta1-induced migration of bone mesenchymal stem cells couples bone resorption with formation. Nat Med 15, 757–765 (2009). 19584867 10.1038/nm.1979 PMC2727637

[b25] Cezar C. A. . Biologic-free mechanically induced muscle regeneration. Proc Natl Acad Sci USA 113, 1534–1539 (2016). 26811474 10.1073/pnas.1517517113 PMC4760832

[b26] Tam K. F., Cheung W. H., Lee K. M., Qin L. & Leung K. S. Shockwave exerts osteogenic effect on osteoporotic bone in an ovariectomized goat model. Ultrasound Med Biol 35, 1109–1118 (2009). 19394753 10.1016/j.ultrasmedbio.2009.01.001

[b27] Chen Y. J. . Shock wave application enhances pertussis toxin protein-sensitive bone formation of segmental femoral defect in rats. J Bone Miner Res 18, 2169–2179 (2003). 14672352 10.1359/jbmr.2003.18.12.2169

[b28] Suhr F. . Cell biological effects of mechanical stimulations generated by focused extracorporeal shock wave applications on cultured human bone marrow stromal cells. Stem Cell Res 11, 951–964 (2013). 23880536 10.1016/j.scr.2013.05.010

[b29] Wang F. S. . Superoxide mediates shock wave induction of ERK-dependent osteogenic transcription factor (CBFA1) and mesenchymal cell differentiation toward osteoprogenitors. J Biol Chem 277, 10931–10937 (2002). 11784711 10.1074/jbc.M104587200

[b30] Chen Y. J. . Activation of extracellular signal-regulated kinase (ERK) and p38 kinase in shock wave-promoted bone formation of segmental defect in rats. Bone 34, 466–477 (2004). 15003794 10.1016/j.bone.2003.11.013

[b31] Hu J. . Focal Adhesion Kinase Signaling Mediated the Enhancement of Osteogenesis of Human Mesenchymal Stem Cells Induced by Extracorporeal Shockwave. Sci Rep 6, 20875 (2016). 26863924 10.1038/srep20875 PMC4750003

[b32] Engler A. J., Sen S., Sweeney H. L. & Discher D. E. Matrix elasticity directs stem cell lineage specification. Cell 126, 677–689 (2006). 16923388 10.1016/j.cell.2006.06.044

[b33] Karageorgiou V. & Kaplan D. Porosity of 3D biomaterial scaffolds and osteogenesis. Biomaterials 26, 5474–5491 (2005). 15860204 10.1016/j.biomaterials.2005.02.002

[b34] Penk A. . The pore size of PLGA bone implants determines the de novo formation of bone tissue in tibial head defects in rats. Magn Reson Med 70, 925–935 (2013). 23165861 10.1002/mrm.24541

[b35] Komori T. Regulation of bone development and extracellular matrix protein genes by RUNX2. Cell Tissue Res 339, 189–195 (2010). 19649655 10.1007/s00441-009-0832-8

[b36] Ducy P. . A Cbfa1-dependent genetic pathway controls bone formation beyond embryonic development. Genes Dev 13, 1025–1036 (1999). 10215629 10.1101/gad.13.8.1025 PMC316641

[b37] Nakashima K. . The novel zinc finger-containing transcription factor osterix is required for osteoblast differentiation and bone formation. Cell 108, 17–29 (2002). 11792318 10.1016/s0092-8674(01)00622-5

[b38] Nishio Y. . Runx2-mediated regulation of the zinc finger Osterix/Sp7 gene. Gene 372, 62–70 (2006). 16574347 10.1016/j.gene.2005.12.022

[b39] Catalano M. G. . Extracorporeal shockwaves (ESWs) enhance the osteogenic medium-induced differentiation of adipose-derived stem cells into osteoblast-like cells. J Tissue Eng Regen Med (2014). 10.1002/term.1922 24889884

[b40] Lee S. J. . Surface modification of 3D-printed porous scaffolds via mussel-inspired polydopamine and effective immobilization of rhBMP-2 to promote osteogenic differentiation for bone tissue engineering. Acta Biomater 40, 182–191 (2016). 26868173 10.1016/j.actbio.2016.02.006

[b41] Crane J. L. & Cao X. Bone marrow mesenchymal stem cells and TGF-beta signaling in bone remodeling. J Clin Invest 124, 466–472 (2014). 24487640 10.1172/JCI70050 PMC3904610

[b42] Zhao L., Jiang S. & Hantash B. M. Transforming growth factor beta1 induces osteogenic differentiation of murine bone marrow stromal cells. Tissue Eng Part A 16, 725–733 (2010). 19769530 10.1089/ten.TEA.2009.0495

[b43] Feng Y. F. . Influence of architecture of beta-tricalcium phosphate scaffolds on biological performance in repairing segmental bone defects. PLoS One 7, e49955 (2012). 23185494 10.1371/journal.pone.0049955 PMC3503864

[b44] Arinzeh T. L. . Allogeneic mesenchymal stem cells regenerate bone in a critical-sized canine segmental defect. J Bone Joint Surg Am 85-A, 1927–1935 (2003). 14563800 10.2106/00004623-200310000-00010

[b45] Quarto R. . Repair of large bone defects with the use of autologous bone marrow stromal cells. N Engl J Med 344, 385–386 (2001). 11195802 10.1056/NEJM200102013440516

[b46] Zhai L. . Effects of Focused Extracorporeal Shock Waves on Bone Marrow Mesenchymal Stem Cells in Patients with Avascular Necrosis of the Femoral Head. Ultrasound Med Biol (2015). 10.1016/j.ultrasmedbio.2015.10.021 26674675

[b47] Chen Y. J. . Recruitment of mesenchymal stem cells and expression of TGF-beta 1 and VEGF in the early stage of shock wave-promoted bone regeneration of segmental defect in rats. J Orthop Res 22, 526–534 (2004). 15099631 10.1016/j.orthres.2003.10.005

[b48] Wang F. S. . Ras induction of superoxide activates ERK-dependent angiogenic transcription factor HIF-1alpha and VEGF-A expression in shock wave-stimulated osteoblasts. J Biol Chem 279, 10331–10337 (2004). 14681237 10.1074/jbc.M308013200

[b49] Gaspar D. A., Gomide V. & Monteiro F. J. The role of perfusion bioreactors in bone tissue engineering. Biomatter 2, 167–175 (2012). 23507883 10.4161/biom.22170 PMC3568103

[b50] Hoshiba T., Kawazoe N., Tateishi T. & Chen G. Development of stepwise osteogenesis-mimicking matrices for the regulation of mesenchymal stem cell functions. J Biol Chem 284, 31164–31173 (2009). 19762920 10.1074/jbc.M109.054676 PMC2781515

[b51] Harada N. . Bone regeneration in a massive rat femur defect through endochondral ossification achieved with chondrogenically differentiated MSCs in a degradable scaffold. Biomaterials 35, 7800–7810 (2014). 24952976 10.1016/j.biomaterials.2014.05.052

[b52] Galmiche M. C., Koteliansky V. E., Briere J., Herve P. & Charbord P. Stromal cells from human long-term marrow cultures are mesenchymal cells that differentiate following a vascular smooth muscle differentiation pathway. Blood 82, 66–76 (1993). 8324235

[b53] Pittenger M. F. . Multilineage potential of adult human mesenchymal stem cells. Science 284, 143–147 (1999). 10102814 10.1126/science.284.5411.143

[b54] Tsutsumi S. . Retention of multilineage differentiation potential of mesenchymal cells during proliferation in response to FGF. Biochem Biophys Res Commun 288, 413–419 (2001). 11606058 10.1006/bbrc.2001.5777

[b55] Shang F. . The effect of licochalcone A on cell-aggregates ECM secretion and osteogenic differentiation during bone formation in metaphyseal defects in ovariectomized rats. Biomaterials 35, 2789–2797 (2014). 24439395 10.1016/j.biomaterials.2013.12.061

[b56] Qin L. . Low intensity pulsed ultrasound increases the matrix hardness of the healing tissues at bone-tendon insertion-a partial patellectomy model in rabbits. Clin Biomech (Bristol, Avon) 21, 387–394 (2006). 10.1016/j.clinbiomech.2005.11.008 16427166

[b57] Zhang G. . A delivery system targeting bone formation surfaces to facilitate RNAi-based anabolic therapy. Nat Med 18, 307–314 (2012). 22286306 10.1038/nm.2617

[b58] Zhang Y. . Implant-derived magnesium induces local neuronal production of CGRP to improve bone-fracture healing in rats. Nat Med 22, 1160–1169 (2016). 27571347 10.1038/nm.4162 PMC5293535

[b59] Qin L. . Multiple bioimaging modalities in evaluation of an experimental osteonecrosis induced by a combination of lipopolysaccharide and methylprednisolone. Bone 39, 863–871 (2006). 16765664 10.1016/j.bone.2006.04.018 PMC7103395

[b60] He Y. X. . Impaired bone healing pattern in mice with ovariectomy-induced osteoporosis: A drill-hole defect model. Bone 48, 1388–1400 (2011). 21421090 10.1016/j.bone.2011.03.720

