# Supplementary material for: An Innovative Approach for Enhancing Bone Defect Healing Using PLGA Scaffolds Seeded with Extracorporeal-shock-wave-treated Bone Marrow Mesenchymal Stem Cells (BMSCs)
Source: Sci Rep. 2017 Mar 8;7:44130. doi: 10.1038/srep44130 (PMC5341040; doi:10.1038/srep44130)
Supplement: Supplementary Information [file srep44130-s1.pdf]

Supplementary Information

An Innovative Approach for Enhancing Bone Defect Healing Using PLGA Scaffolds Seeded with Extracorporeal-shock-wave-treated Bone Marrow Mesenchymal Stem Cells (BMSCs)

Youbin Chen[1,a], Jiankun Xu[1,2,a], Zhonglian Huang[1,a], Menglei Yu[3] , Yuantao Zhang[1], Hongjiang Chen[1], Zebin Ma[1], Haojie Liao[1], Jun Hu [1,*]

Affiliations:

[1]Department of Orthopedics, First Affiliated Hospital, Shantou University Medical College, 57 Changping Road, Shantou, Guangdong 515041, China.

[2]Department of Orthopaedics and Traumatology, Faculty of Medicine, the Chinese University of Hong Kong, Hong Kong SAR 999077, China .

[3]Guangdong Provincial Key Laboratory of Malignant Tumor Epigenetics and Gene Regulation, Emergency Department, Sun Yat-Sen Memorial Hospital, Sun Yat-Sen University, Guangzhou, Guangdong 510120, China.

[a] These authors contributed equally to this work.

[*] To whom correspondence should be addressed: Jun Hu, the Department of Orthopaedics, the First Affiliated Hospital, Shantou University Medical College, 57 Changping Rd., Shantou, Guangdong 515041, China, Tel.: +86-754-88905206; Fax: +86-754-88259850; E-mail: hjzkm@vip.163.com

Supplementary figure 1

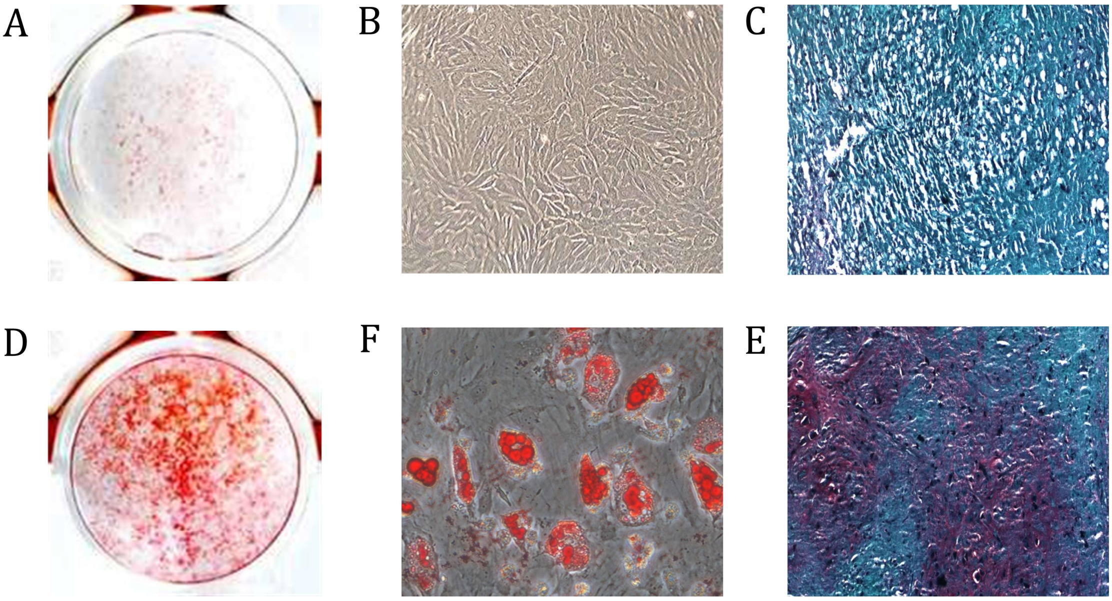

Figure S1. Rat bone marrow-derived cells cultured in osteogenic induction medium differentiated into osteoblasts as confirmed by 1% Alizarin red S staining; calcium nodules stained red (D). BMSC differentiation to adipocytes in adipogenic induction medium was verified by 0.18% Oil Red O staining (E). 0.05% Safranine O staining (F) showed that BMSCs differentiated into chondrocytes in chondrogenic induction medium. All staining have an negative control (A, B, C).
